# The role and machine learning analysis of mitochondrial autophagy-related gene expression in lung adenocarcinoma

**DOI:** 10.3389/fimmu.2025.1509315

**Published:** 2025-04-17

**Authors:** Binyu Wang, Di Liu, Danfei Shi, Xinmin Li, Yong Li

**Affiliations:** ^1^ Department of Clinical Laboratory, The First Affiliated Hospital of Huzhou University, The First People’s Hospital of Huzhou City, Huzhou, Zhejiang, China; ^2^ Department of Clinical Laboratory, Huzhou Maternity & Child Health Care Hospital, Huzhou, Zhejiang, China; ^3^ Department of Pathology, The First Affiliated Hospital of Huzhou University, The First People’s Hospital of Huzhou City, Huzhou, Zhejiang, China; ^4^ Department of Clinical Laboratory, Chongqing Hospital of Traditional Chinese Medicine, ChongQing, China

**Keywords:** lung adenocarcinoma, gene, machine learning, mitochondrial autophagy, WGCNA

## Abstract

**Objective:**

Lung adenocarcinoma (LUAD) continues to be a primary cause of cancer-related mortality globally, highlighting the urgent need for novel insights finto its molecular mechanisms. This study aims to investigate the relationship between gene expression and mitophagy in LUAD, with an emphasis on identifying key biomarkers and elucidating their roles in tumorigenesis and immune cell infiltration.

**Methods:**

We utilized datasets GSE151101 and GSE203609 from the Gene Expression Omnibus (GEO) database to identify differentially expressed genes (DEGs) associated with lung cancer and mitophagy. DEGs were identified using GEO2R, filtered based on criteria of P < 0.05 and log2 fold change ≥ 1. Subsequently, Weighted Gene Co-expression Network Analysis (WGCNA) was conducted to classify DEGs into modules. Functional annotation of these modules was performed using Gene Ontology (GO) and Kyoto Encyclopedia of Genes and Genomes (KEGG) pathway analyses. Gene Set Enrichment Analysis (GSEA) was applied to the most relevant module, designated as the greenyellow module. To identify critical biomarkers, machine learning algorithms including Random Forest, Least Absolute Shrinkage and Selection Operator (LASSO) regression, and Support Vector Machine (SVM) were employed. Validation of the findings was conducted using The Cancer Genome Atlas (TCGA) database, Human Protein Atlas (HPA), quantitative PCR (qPCR), and immune cell infiltration analysis via CIBERSORTx.

**Results:**

Our analysis identified 11,012 overlapping DEGs between the two datasets. WGCNA revealed 11 modules, with the green-yellow module exhibiting the highest correlation. Functional enrichment analysis highlighted significant associations with FOXM1 signaling pathways and retinoblastoma in cancer. Machine learning algorithms identified COASY, FTSJ1, and MOGS as pivotal genes. These findings were validated using TCGA data, qPCR experiments, which demonstrated high expression levels in LUAD samples. Immunohistochemistry from HPA confirmed consistency between protein levels and RNA-seq data. Furthermore, pan-cancer analysis indicated that these genes are highly expressed across various cancer types. Immune infiltration analysis suggested significant correlations between these genes and specific immune cell populations.

**Conclusion:**

COASY, FTSJ1 and MOGS have emerged as critical biomarkers in LUAD, potentially influencing tumorigenesis through mitophagy-related mechanisms and immune modulation. These findings provide promising avenues for future research into targeted therapies and diagnostic tools, thereby enhancing LUAD management.

## 1 Introduction

Lung cancer is the leading cause of cancer-related mortality worldwide, accounting for approximately 25% of all cancer deaths. Among these, lung adenocarcinoma (LUAD) represents 40% of lung cancer cases (1). Despite significant advances in medical research, the prognosis for LUAD patients remains poor, with a five-year survival rate of only 19% (2). The primary risk factors for LUAD include smoking, exposure to air pollution, and genetic predispositions (3). While current diagnostic methods such as low-dose computed tomography (LDCT) have improved early detection rates, they are also associated with high false-positive rates and potential over-diagnosis (4). Consequently, there is an urgent need for more accurate and reliable diagnostic and therapeutic strategies.

LUAD originates from precancerous lesions and gradually progresses to invasive adenocarcinoma (5). However, we still have limited knowledge regarding the cellular heterogeneity and molecular events during the development process. Wang Z et al. conducted single-cell RNA sequencing on 268,471 cells from 25 LUAD patients and discovered that as LUAD progresses, a group of cells similar to alveolar type 2 cells gradually exhibit characteristics of stem cell-like cells, strongly expressing ribosomal and mitochondrial genes that promote tumor progression. Among them, MDK and TIMP1 are upregulated in the early stages of LUAD and may contribute to disease progression, which can serve as potential biomarkers and therapeutic targets for understanding the pathogenesis of LUAD (6). Additionally, multiple recent studies have identified biomarkers of significant value in aspects such as early diagnosis, immunotherapy, and prognosis assessment through big data analysis and experimental exploration, such as PANX1, B4GALT1 and circRNA-002178 (7–9). Therefore, it’s beneficial to operate data analysis and verification of the genomic profile of LUAD, as it can provide more possibilities to search for new diagnostic biomarkers and therapeutic strategies for LUAD.

Mitochondrial autophagy, or mitophagy, is a selective form of autophagy that specifically targets damaged or superfluous mitochondria for degradation (10). This process is crucial for maintaining cellular homeostasis and has been implicated in various diseases, including cancer (11). In LUAD, dysregulated mitophagy has been observed, indicating a potential link between mitochondrial dysfunction and tumorigenesis (12). Research have shown that key regulators of mitophagy, such as PINK1 and Parkin, are frequently altered in cancer cells, resulting in abnormal mitochondrial dynamics and metabolic reprogramming (13). These findings underscore the therapeutic potential of targeting mitophagy in LUAD.

In addition to LUAD, dysregulated mitophagy has been investigated in various other cancers, including breast and liver cancer (14). In breast cancer, mitophagy-related genes have been identified as differentially expressed, which correlates with disease progression and patient outcomes (15). Likewise, in liver cancer, the inhibition of mitophagy has been associated with enhanced tumor growth and metastasis (16). These findings highlight the critical role of mitophagy in cancer biology and its potential utility as a biomarker for both diagnosis and prognosis.

Our research aims to investigate the gene expression profiles associated with mitophagy in LUAD. We utilized datasets from the Gene Expression Omnibus (GEO) database, including GSE151101 for lung cancer and GSE203609 for mitophagy-related data. Differentially expressed genes (DEGs) were identified using GEO2R and R software. Subsequently, Weighted Gene Co-expression Network Analysis (WGCNA) was conducted to determine key gene modules involved in mitophagy. To elucidate the biological significance of these genes, we performed functional enrichment analyses, including Gene Ontology (GO), Kyoto Encyclopedia of Genes and Genomes (KEGG), and Gene Set Enrichment Analysis (GSEA). Machine learning algorithms, such as Random Forest, LASSO regression, and SVM, were employed to identify potential biomarkers. The findings were validated using data from The Cancer Genome Atlas (TCGA) and Human Protein Atlas (HPA) databases, and further confirmed by quantitative real-time PCR (qPCR) experiments.

In conclusion, this study endeavors to provide an in-depth understanding of the role of mitophagy in LUAD and identify potential biomarkers for early diagnosis and targeted therapy. Through the integration of bioinformatics analyses and experimental validation, we aim to contribute to the development of more effective diagnostic and therapeutic strategies for LUAD.

## 2 Method

### 2.1 Data source

Query the GEO database for data related to “lung cancer” and identify dataset GSE151101 (17). Details of the dataset are as follows:

GSE151101 data set:

Platform: GPL11532 [HuGene-1_1-st] Affymetrix Human gene 1.1ST chip [Transcript (gene) version].

Specimens: A total of 124 lung cancer specimens and 113 non-lung cancer specimens were analyzed.

This dataset offers a comprehensive resource for gene expression analysis of both lung and non-lung cancer specimens, thereby facilitating further research in this field. We also retrieved data pertaining to “mitochondrial autophagy” and identified the GSE203609 dataset (18). The specifics of this dataset are as follows:

Platform: GPL16791 Illumina HiSeq 2500 (Sapiens).

Samples: 3 cases of TBHP + ML treatment group, 3 cases of TBHP treatment group and 3 cases of control group.

**Figure 1:**
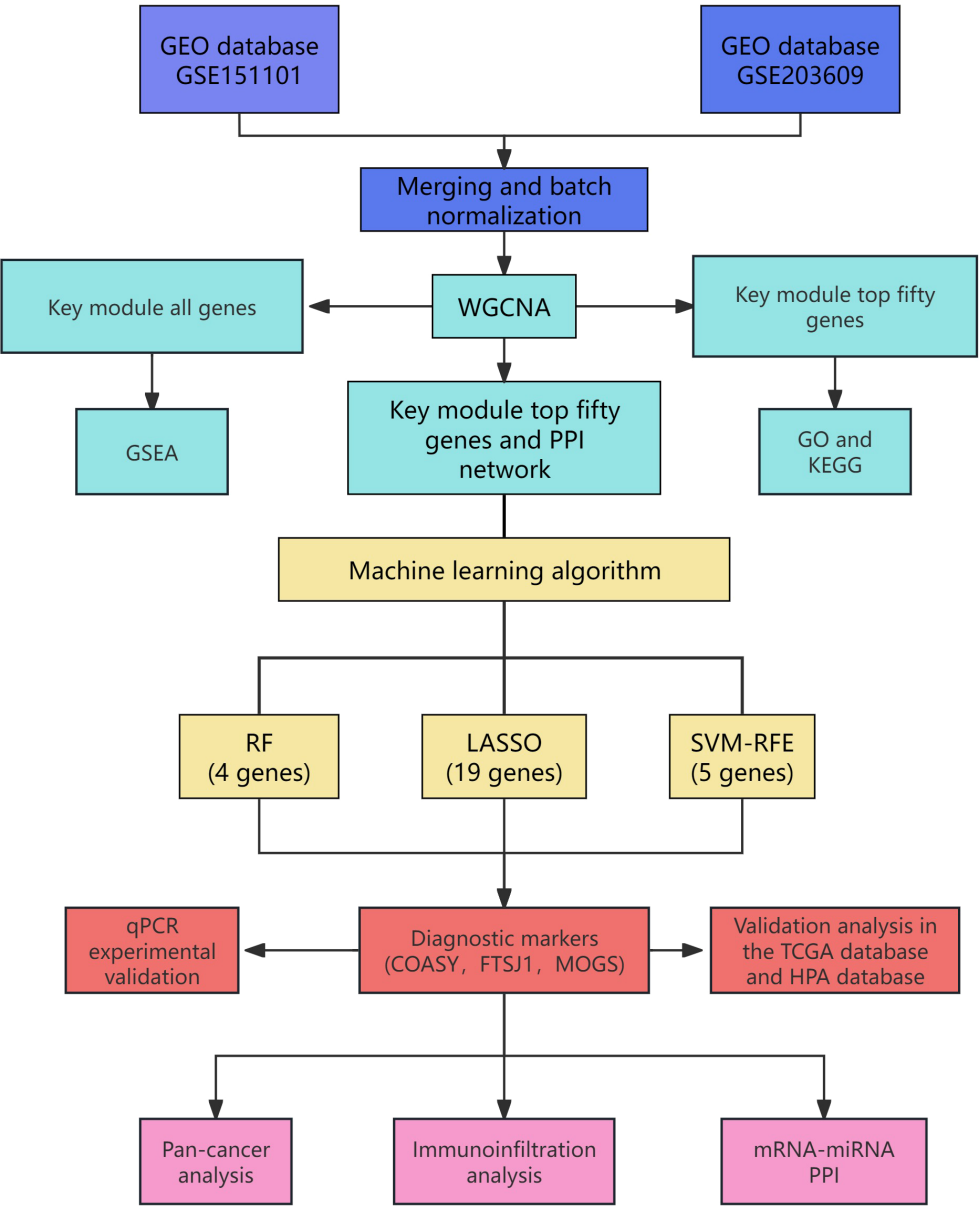
Project flow chart.

This dataset offers crucial insights into the gene expression analysis of mitochondrial autophagy and facilitates subsequent research endeavors. As illustrated in **Figure 1**, the flow diagram outlines the project’s methodology.

### 2.2 DEG data processing

We extracted GEO data using the GEO2R tool (19) and filtered the data using R software (version 4.2.1) (20) with the screening criteria of *P* < 0.05 and log2FC ≥ 1. Subsequently, we generated heatmaps using the R package “ggplot2” (version 3.3.6) to visualize the expression patterns of the identified DEGs. Additionally, Venn diagrams were created using VENNY (version 2.1) to illustrate the overlap of DEGs.

### 2.3 WGCNA enrichment analysis of DEGs

We performed WGCNA analysis of the expression levels of these DEGs in the TCGA LUAD dataset using the WGCNA (version 1.73) software package in R (version 4.2.1) (21). Data visualization is generated by the R package ggplot2 (version 3.3.6).

### 2.4 GO, KEGG and GSEA enrichment analysis of Green-yellow module

To elucidate the functional significance of genes in the green-yellow module, we conducted GO annotation (22) and KEGG pathway analysis (23) on the top 50 genes. The GO annotation was performed using the DAVID tool (version 6.7) (24) to classify and identify the functions of these genes and their products. KEGG pathway analysis was employed to explore signal transduction pathways associated with these genes. Additionally, we performed GSEA analysis on all genes within the green-yellow module using R software (version 4.2.1). The standard of statistical significance was adjusted for *P* < 0.05.

### 2.5 Machine learning algorithm

We use Random Forest (25), Least Absolute Shrinkage and Selection Operator (LASSO) Regression (26), and Support Vector Machine (SVM) (27) to identify key biomarkers. In random forest analysis (R package: randomForest[4.7.1.1]), we use its high accuracy to identify and select the most important feature variables by constructing multiple decision trees; The ranking of important genes is obtained through SVM analysis (R package: e1071[1.7.13]) (28), which is good at handling nonlinear classification problems and can reduce the impact caused by the number of samples; Then, the regularization property of LASSO (R package: glmnet[4.1.7]) regression was used to further screen key features, providing higher interpretability for the analysis results.These methods collectively helped us identify three key target genes (COASY, FTSJ1, and MOGS) and ensure that the model is more concise and effective in prediction. By integrating multiple methods, we have improved the stability and interpretability of the model.

In addition, we describe statistical methods for evaluating identified biomarkers, including specific test methods and multiple hypothesis test corrections. For example, in the significance analysis, we used the student t test and Wilcoxon rank sum test to compare differences in gene expression and used FDR correction to control for false positive rates.

### 2.6 LUAD data from TCGA database

We obtained and organized RNAseq data from the TCGA-LUAD project (https://portal.gdc.cancer.gov) and used R language software (version 4.2.1) (21) to analyze the expression level, survival curve and ROC curve of target genes in LUAD. ROC analysis uses the pROC package (version 1.18.0) and data visualization uses the ggplot2 package (version 3.3.6).

### 2.7 Expression of pivot gene protein in human protein map and HPA database validation

We assessed the expression levels and distribution of three gene proteins in LUAD specimens and non-cancer samples from the Human Protein Atlas (HPA) public database.

### 2.8 Quantitative polymerase chain reaction

QPCR was used to detect the expression levels of COASY, FTSJ1 and MOGS in LUAD. We recruited 12 patients with LUAD who were admitted between January 1, 2024 and January 31, 2024. Additionally, 12 healthy individuals who underwent routine health examinations during the same period were included as the control group. The inclusion criteria for LUAD patients were as follows: (1) Age ≤ 90 years; (2) No history of chemoradiotherapy; (3) No fever or infection within 3 months before blood collection; (4) No history of blood transfusion.

QPCR tests were configured in accordance with the guidelines outlined in the MIQE Guide. Three genes were selected to validate the RNA-seq results. QPCR primers were designed using Primer3 software (http://bioinfo.ut.ee/primer3-0.4.0/) and synthesized by Sangon Biotechnology Co., Ltd. (Shanghai, China). For cDNA synthesis, 1Mg of total RNA was reverse-transcribed using the PrimeScript RT reagent kit (TakaraBio™Inc., SAN Jose, CA) according to the manufacturer’s protocol. Quantitative RT-PCR was performed on the CFX96 Real-Time PCR system (Bio-RAD Laboratories, Hercules, CA, USA) using TB Green Premixed Ex Taq II (Takara Bio Inc.). The consumables used included eight PCR tubes from Axygen^®^ brand products (Corning Corporation, Corning, New York, USA). Each quantification was conducted using a 25ML reaction mixture containing 12.5ML TB Green Premix Ex Taq II, 1ML (10MM) of each primer, 8.5ML RNase-free water, and 2Ml of 1:5 diluted cDNA. PCR amplification conditions consisted of initial denaturation at 95°C for 30 s, followed by 40 cycles of denaturation at 95°C for 5 s and annealing at 60°C for 30 s. After cooling to 65°C for 5s, the melting curve at the end of each PCR was obtained by gradually increasing the temperature to 95°C (with an incremental rate of 0.5°C/s). All samples underwent identical amplification analysis, eliminating the need for successive calibration. The data obtained were analyzed using Bio-Rad CFX Manager software (version 3.0), which generates raw quantitative cycle (Cq) values for each reaction using the 2-ΔΔCT method. Further details of qPCR can be found in the MIQE checklist. Primer pairs are used in the following order:

COASY: forward primer, 5’-CTTGAGAATGACCTGGAGGAACTTG-3’, and reverse primer, 5’-GCCAGTCAGCCCAATTACATAGAG-3’.

FTSJ1: forward primer, 5’-GCTCCTGATGGCTCTGAACATTG-3’, and reverse primer, 5’-AGCACGCTGGAGAAGAAGACC-3’.

MOGS: forward primer, 5’-GGCAGTTCTTGATACAGCAGGTG-3’, and reverse primer, 5’-GTCTTGGCAGGGCTTGATTTCC-3’.

### 2.9 Analysis of infiltration of immune cells

The Sieber sorting algorithm was applied to the GSE151101 dataset to determine the significant association between the target gene and various immune cells through correlation analysis. All analysis and visualization were performed in R software (4.2.1) (21). Based on CIBERSORTx website (https://cibersortx.stanford.edu/) access to 22 immune cells, using codon sequence. R (script) analysis core algorithm (29) to detect the gene expression profile of signature matrix. Presentation data acquisition: RNAseq data is downloaded from the TCGA-LUAD project STAR process of the TCGA database and extracted in TPM format and clinical data format.

### 2.10 Pan-cancer analysis and miRNA analysis of COASY, FTSJ1, and MOGS

In this study, we used R software (version 4.2.1) (21) and R packages ggplot2 [3.3.6], stats [4.2.1] and car [3.1-0], and TCGA database to analyze mRNA expression of COASY, FTSJ1 and MOGS (30). Data processing method: log2 (value +1). In addition, we also analyzed the mirnas corresponding to these three target genes. MiRNAs is retrieved from three databases: TargetScan (31), ENCORI (32), and miRwalk (33). Venn diagram and protein-protein interaction (PPI) network were constructed for further analysis.

### 2.11 Ethics

The research protocol has been approved by the Medical Ethics Committee of the Medical Research and Clinical Trial Ethics Committee of Huzhou First People’s Hospital (approval number: 2023KYLL014). All participants provided written informed consent prior to their involvement in the study.

## 3 Result

### 3.1 Identification of differentially expressed genes

**Figure 2:**
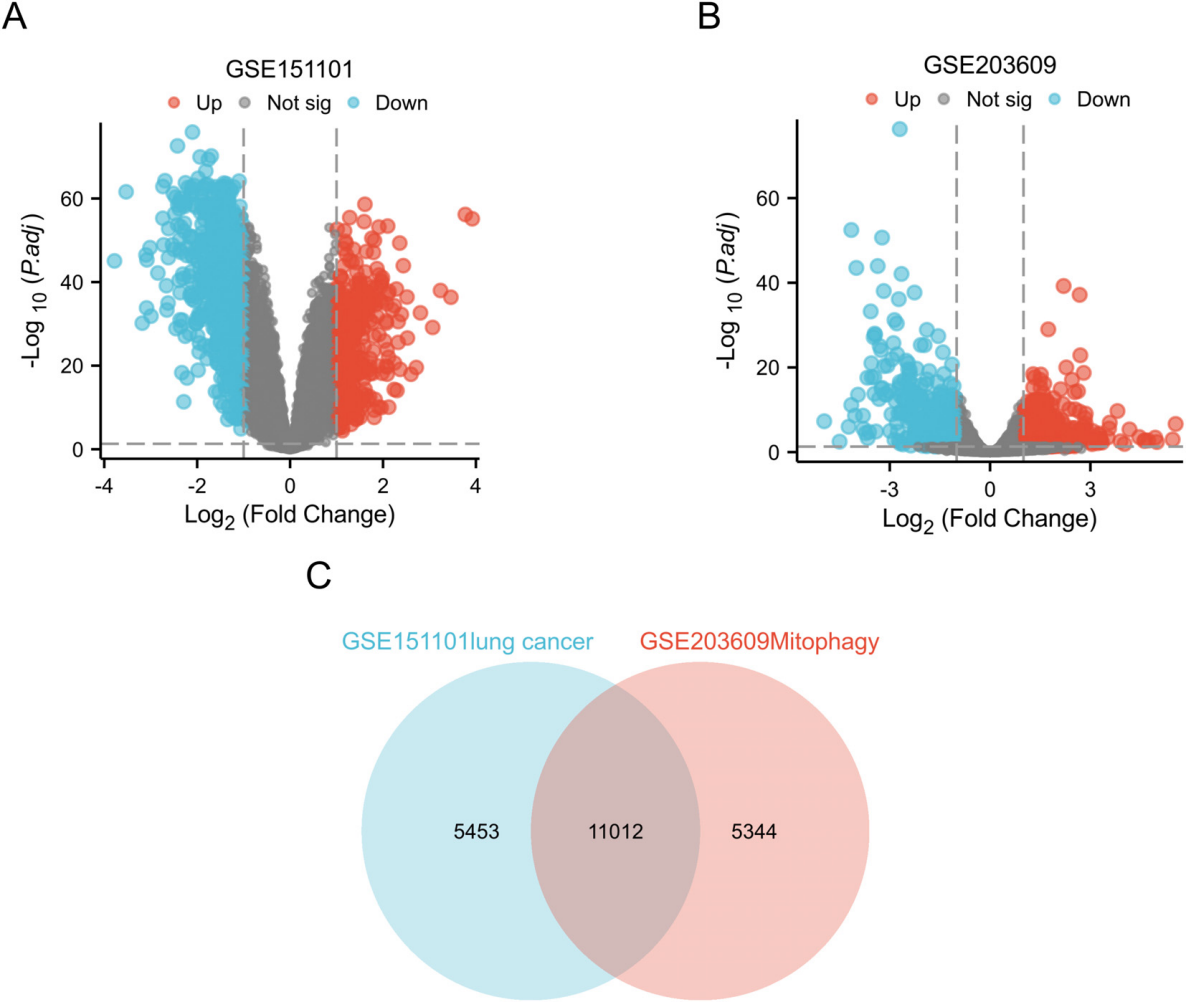
Identification of DEGs in two GEO datasets. **(A)** In the GSE151101 dataset, DEGs volcano maps describing the differences in expression between LUAD specimens and normal lung specimens; **(B)** In dataset GSE203609, DEGs volcano maps with differences in expression between TBHP+ML treatment group and TBHP treatment are described; **(C)** Venn diagram highlights 11012 overlapping DEGs in the GSEGSE151101 and GSE203609 datasets.

The gene expression data were processed and normalized using standard log FC > 1 and *P* < 0.05. DEGs were subsequently identified in both datasets using the GEO2R online tool (**Figure 2**).

### 3.2 WGCNA enrichment analysis of DEGs

**Figure 3:**
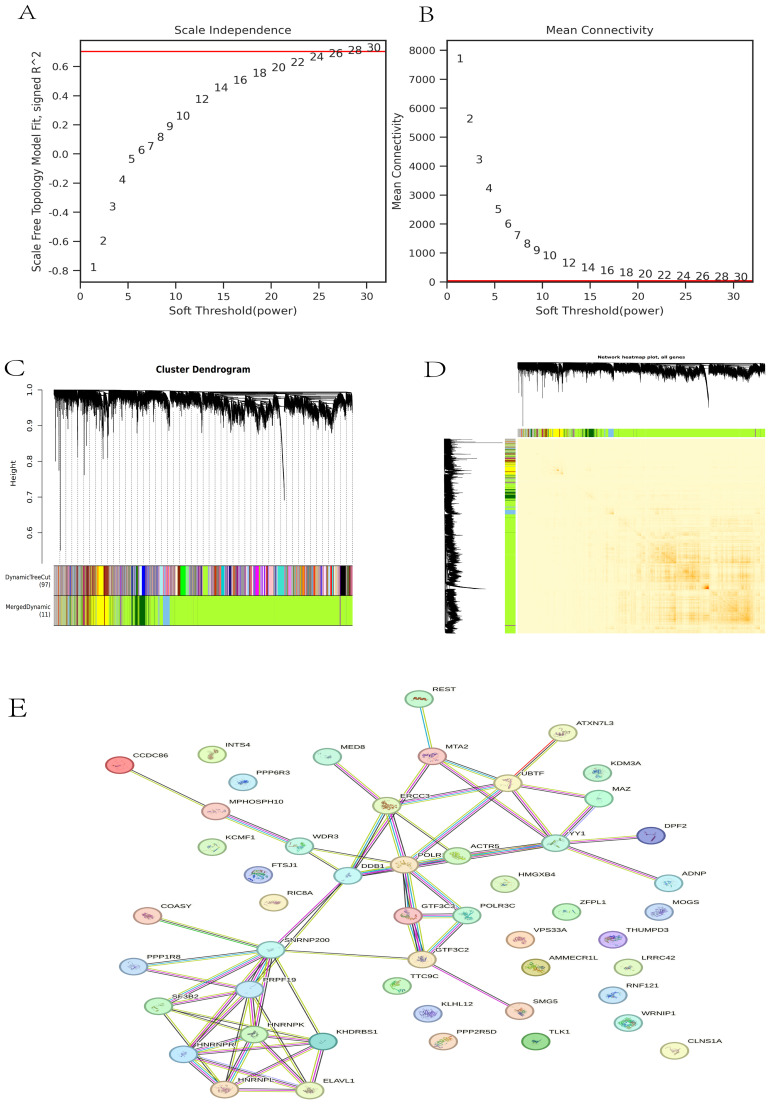
WGCNA analysis. **(A)** The scale independence of DEGs; **(B)** The correlation of each gene module; **(C)** Gene tree and gene module; **(D)** Cluster heat map of DEGs; **(E)** PPI map of the top 50 genes in the green-yellow module.

The gene co-expression network is a scale-free weighted gene network. To better adhere to the power-law distribution characteristic of scale-free networks, it is crucial to select an appropriate power for the adjacency matrix weight parameter. In this analysis, a power value of 30 was chosen (**Figure 3A**). Based on this selected power values, a weighted co-representation network model was constructed, and DEGs were clustered into 11 modules (**Figure 3B**). The Grey module comprises genes that do not fit into any specific module and thus lack significant biological relevance, whereas the green-yellow module exhibits the highest correlation among the biologically meaningful modules (**Figures 3C, D**). Subsequently, we utilized the STRING database to construct a PPI network for the top 50 genes within the green-yellow module, thereby facilitating a deeper exploration of potential relationships between DEGs and identifying key genes (**Figure 3E**).

### 3.3 GSEA analysis and GOKEGG analysis of green-yellow module

**Figure 4:**
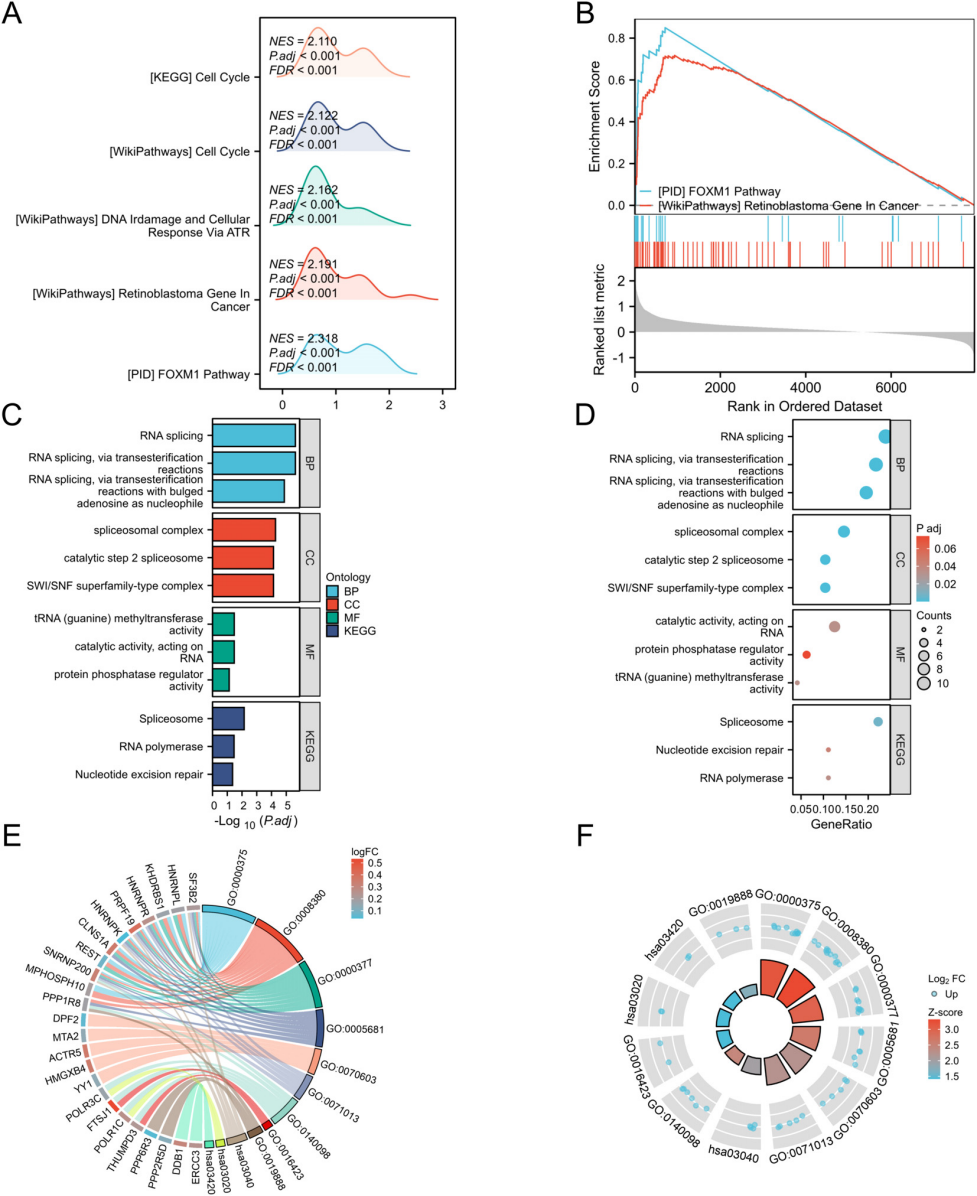
GSEA and GOKEGG analysis of genes in the green-yellow module. **(A)** Mountain maps analyzed by GSEA; **(B)** The classic graph analyzed by GSEA; **(C)** BP, biological process; CC, cell component; MF, molecular function; **(D)** KEGG pathway; **(E)** GOKEGG chord diagram analysis of genes in the green-yellow module; **(F)** GOKEGG circular graph analysis of genes in green-yellow module; All enrichment paths were generated using the ggplot2 package in R.

To gain a deeper understanding of the genes in the green-yellow module, we conducted functional analysis of all genes within this module. Gene Set Enrichment Analysis (GSEA) revealed that these genes were significantly associated with the FOXM1 pathway and retinoblastoma in cancer (**Figures 4A, B**). Then we performed GO and KEGG analyses on the top fifty genes in this module, yielding the following results (**Figures 4C–F**):

Biological processes (BP): RNA splicing, RNA splicing via transesterification, RNA splicing via transesterification with expanded adenosine as a nucleophile;

Cellular component (CC): spliceosome complex, SWI/SNF superfamily complex, catalytic step 2 spliceosome;

Molecular function (MF): catalytic activity of RNA, tRNA (guanine) methyltransferase activity, protein phosphatase regulatory activity;

KEGG pathway: spliceosome, ribonucleic acid polymerase, nucleotide excision repair.

These findings highlight multiple functional roles and pathways associated with genes in the green-yellow module.

### 3.4 The machine learning algorithm selects the target gene

**Figure 5:**
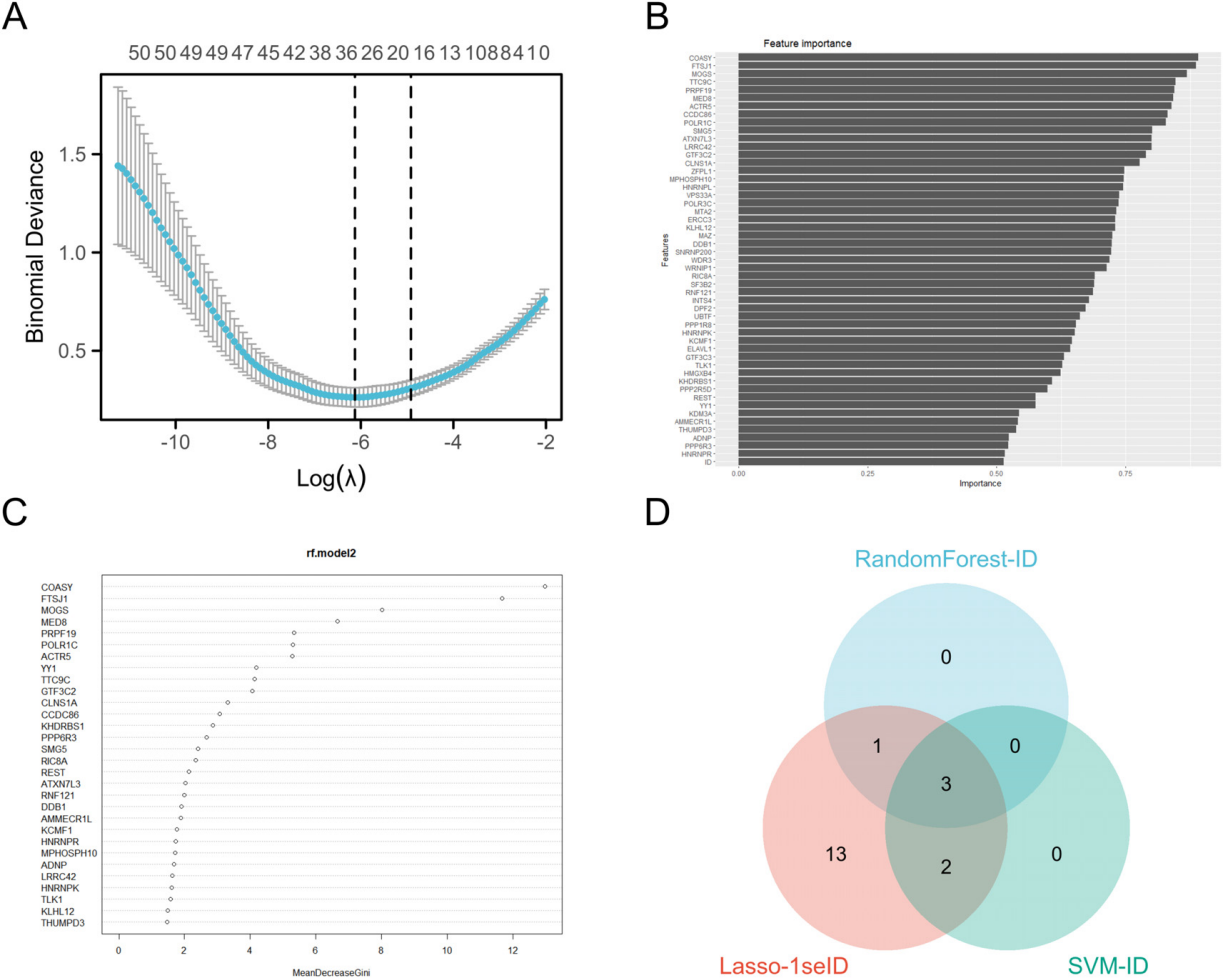
Machine learning algorithms select target genes. **(A)** LASSO analysis coefficient varies with λ parameter; **(B)** SVM analysis results visualization; **(C)** Ranking of important features in the random forest model; **(D)** The three analyses intersect Wayne diagrams.

We conducted Random Forest, SVM and LASSO regression analyses on the top 50 genes in the green-yellow module. In the Random Forest analysis, the four most important characteristic variables of all genes were COASY, FTSJ1, MOGS and MED8, with COASY being the most influential gene among all characteristic variables (**Figure 5C**). For the SVM analysis, the top five genes of importance were COASY, FTSJ1, MOGS, TTC9C and PRPF19 (**Figure 5B**). The LASSO regression analysis identified a broader set of important genes, including PPP6R3, COASY, PRPF19, CLNS1A, MOGS, DPF2, FTSJ1, WDR3, TTC9C, POLR1C, MED8, SNRNP200, ACTR5, PPP1R8, YY1, KLHL12, DDB1, ELAVL1, LRRC42 (**Figure 5A**). Subsequently, we constructed a Venn diagram to identify overlapping genes across the three methods, revealing that COASY, FTSJ1, and MOGS were consistently highlighted as key targets (**Figure 5D**).

As a coenzyme A synthetase, COASY plays a crucial role in cellular energy metabolism and fatty acid synthesis. Aberrant expression of COASY may result in metabolic disturbances, thereby promoting the onset and progression of tumors (34). The protein encoded by FTSJ1 plays a crucial role in rRNA modification, and its overexpression has been associated with increased aggressiveness and poor prognosis in various cancers (35). MOGS, an important endoplasmic reticulum glycosidase, is often overexpressed in tumor cells, contributing to aberrant glycoprotein modification and tumor malignancy (36). These genes are key candidates with significant roles in the biological systems studied, particularly in LUAD (**Figure 5**).

### 3.5 The three target genes were verified by TCGA and qPCR

**Figure 6:**
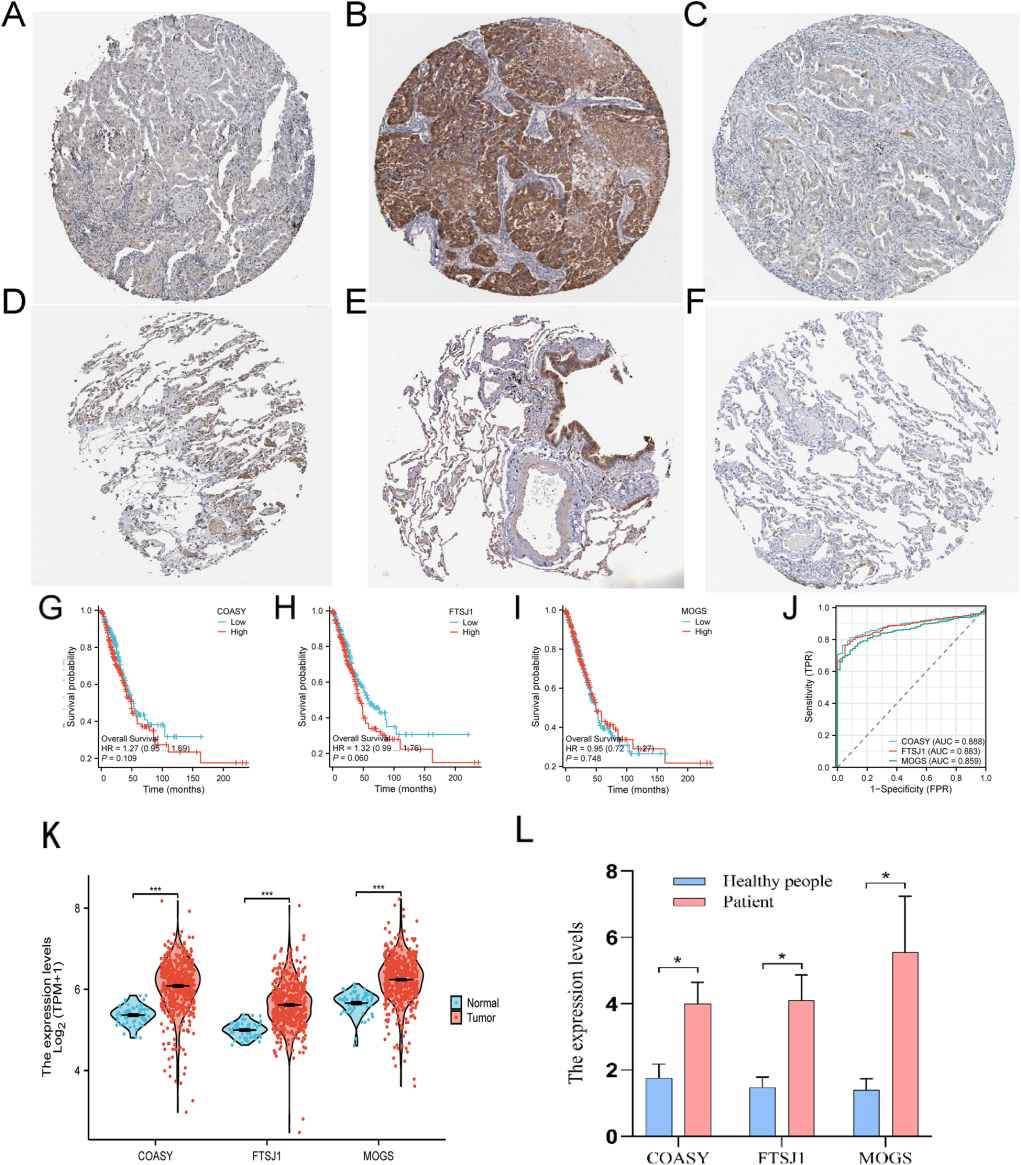
Expression of three target genes COASY, FTSJ1 and MOGS in LUAD. **(A)** Tissue expression of COASY - LUAD tissue staining - human protein profile; **(B)** Tissue expression of FTSJ1 - LUAD tissue staining - human protein profile; **(C)** Tissue expression of MOGS - LUAD tissue staining - human protein profile; **(D)** Tissue expression of COASY - staining of normal parts of the lung - human protein profile; **(E)** Tissue expression of FTSJ1 - Staining of normal parts of the lung - human protein profile; **(F)** Tissue expression of MOGS - staining of normal parts of the lung - human protein profile; **(G–I)** survival curves of these three genes in LUAD; **(J)** ROC curves of these three genes in LUAD; **(K)** The three target genes were verified using the TCGA database; **(L)** These three target genes were verified by qPCR assay (n=8, *P* < 0.05) * P<0.05, *** P <0.001..

In this study, IHC staining in the HPA database showed reduced levels of COASY, FTSJ1, and MOGS proteins in LUAD tissue, consistent with previous findings (**Figures 6A–F**). In addition, we retrieved and organized RNA-seq data from the TCGA-LUAD project (https://portal.gdc.cancer.gov), using concatenated transcripts for comparison to reference (STAR) pipelines available in the TCGA database. The data were normalized to transcripts per million (TPM). Subsequently, we used R language software (version 4.2.1) (21) to analyze survival curves, ROC curves and the expression levels of three target genes—COASY, FTSJ1 and MOGS—in LUAD patients. Our results indicate that LUAD patients with high expression levels of COASY and FTSJ1 had significantly lower survival rates after 50 months compared to those with low expression levels (**Figures 6G–I**). Furthermore, the expression levels of COASY, FTSJ1 and MOGS in LUAD patients were significantly higher than those in normal controls, and the differences were statistically significant (*P* < 0.001, **Figure 6K**). The ROC curve analysis also demonstrated the diagnostic value of these three target genes for LUAD, with AUC values of 0.888 for COASY, 0.883 for FTSJ1, and 0.859 for MOGS (**Figure 6J**). Furthermore, we collected whole blood samples from eight LUAD patients and eight healthy individuals who underwent physical examination in our hospital for qPCR test verification. The results showed that the expression levels of the three target genes were significantly higher in LUAD patients compared to the healthy controls (*P* < 0.05, **Figure 6L**). This comprehensive analysis provides insights into the expression patterns and potential diagnostic and prognostic value of these genes in LUAD (**Figure 6**).

*P < 0.05, ***P < 0.001.

### 3.6 Pan-cancer analysis of COASY, FTSJ1, and MOGS

**Figure 7:**
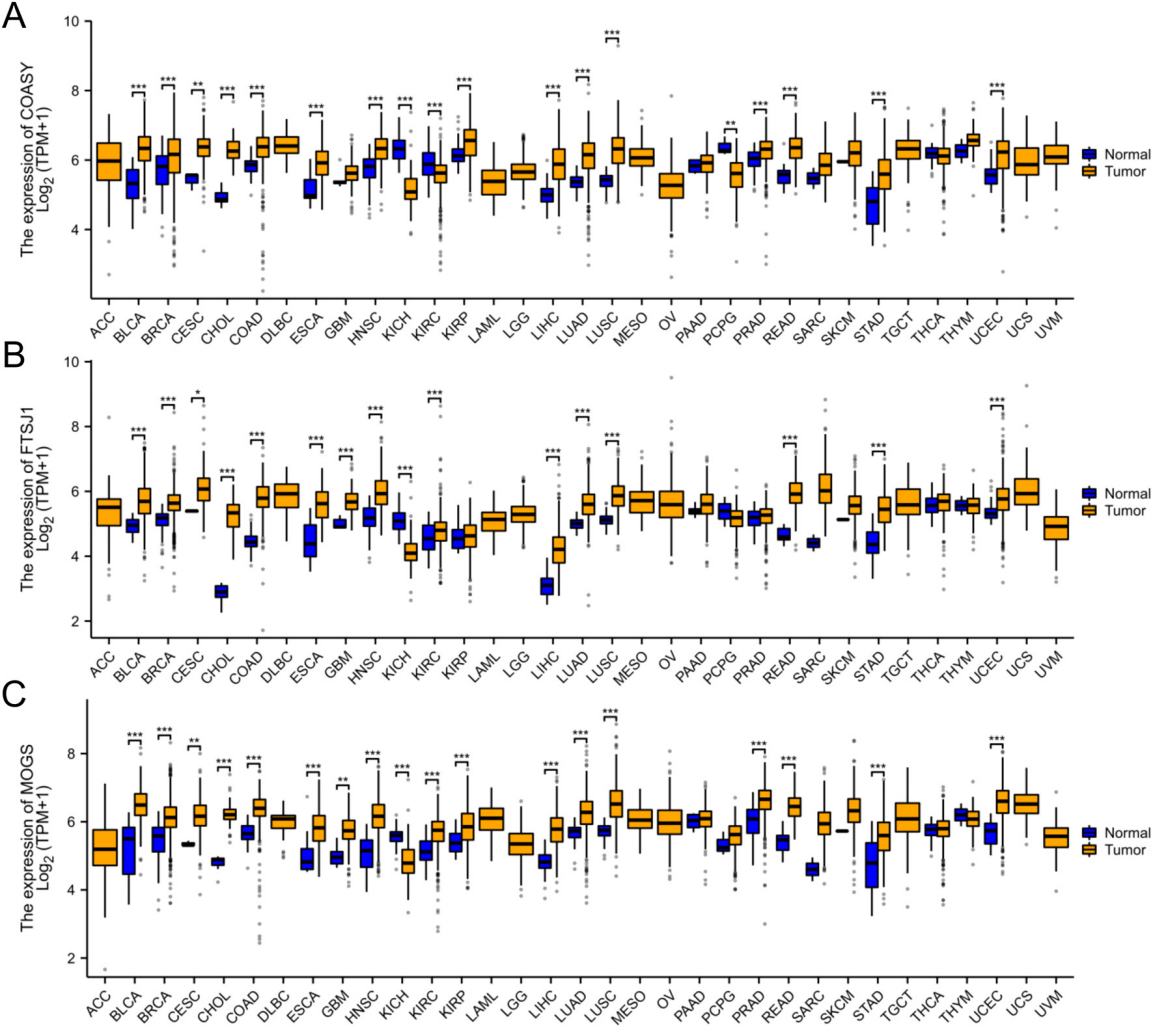
Pan-cancer analysis of COASY, FTSJ1, and MOGS. **(A)** Pan-cancer analysis of COASY; **(B)** Pan-cancer analysis of FTSJ1; **(C)** Pan-cancer analysis of MOGS. * P<0.05, ** P<0.01, *** P<0.001.

In this study, we conducted a pan-cancer analysis of COASY, FTSJ1, and MOGS across various cancer types using the TCGA database (https://portal.gdc.cancer.gov). The analysis was performed using R software (version 4.2.1) (21) and ggplot2 software package. Our results demonstrated that COASY, FTSJ1 and MOGS were significantly overexpressed in 15 types of cancer: BLCA, BRCA, CESC, CHOL, COAD, ESCA, HNSC, KIRP, LIHC, LUAD, LUSC, PRAD, READ, STAD, and UCEC. These observations suggest that COASY, FTSJ1, and MOGS may function as potential tumor promoting genes in cancers with elevated expression levels (**Figure 7**).

### 3.7 Analysis of infiltration of immune cells

**Figure 8:**
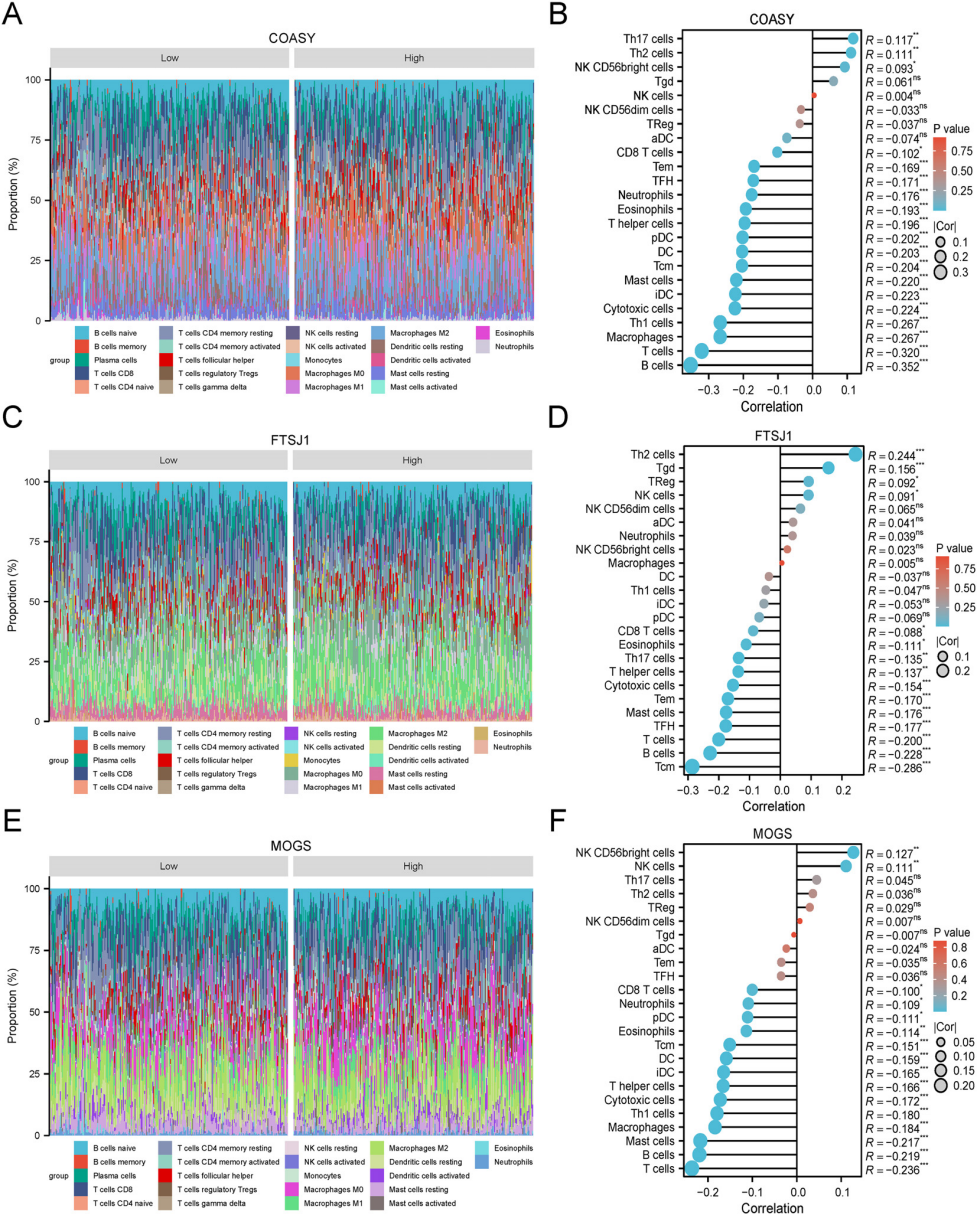
Immune-related infiltration analysis of three target genes. **(A)** COASY-CIBERSORT algorithm-superimposed histogram of LUAD; **(B)** COASY-ssGSEA algorithm-lollipop chart of immunoinfiltration correlation in LUAD; **(C)** FTSJ1-CIBERSORT algorithm-LUAD superimposed histogram; **(D)** FTSJ1-ssGSEA algorithm-lollipop chart of immunoinfiltration correlation in LUAD; **(E)** MOGS-CIBERSORT algorithm-LUAD superimposed histogram; **(F)** MOGS-ssGSEA algorithm-lollipop chart of immunoinfiltration correlation in LUAD.

The Cy-Sort algorithm was applied to the GSE151101 dataset, and correlation analysis showed that COASY, FTSJ1 and MOGS exhibited significant correlations with multiple immune cell types. These findings suggest a potential association between these target genes and the immune cell composition within the LUAD microenvironment. The correlation analyses provide valuable insights into the interactions between these genes and the immune system in the LUAD environment (**Figure 8**).

### 3.8 MiRNA analysis of COASY, FTSJ1 and MOGS

**Figure 9:**
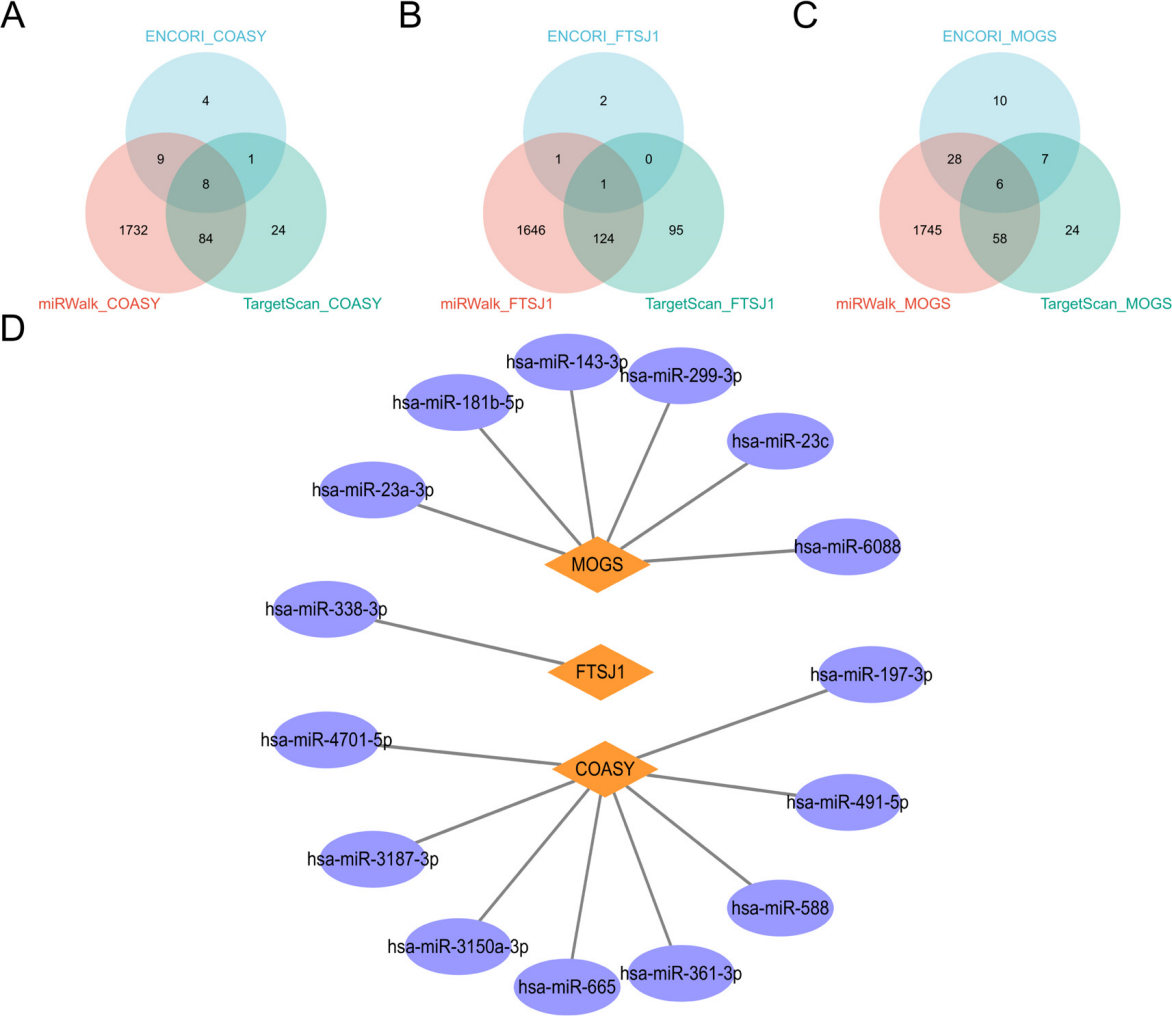
miRNA analysis of COASY, FTSJ1 and MOGS. **(A–C)** These three miRNA databases correspond to the miRNA Venn maps of COASY, FTSJ1, and MOGS, respectively; **(D)** PPI network diagram.

In this study, we comprehensively investigated the microRNA (miRNA) regulatory networks associated with COASY, FTSJ1, and MOGS. By integrating data from three well-established miRNA databases— TargetScan (31), ENCORI (32) and miRwalk (33) —we identified potential miRNAs that regulate COASY, FTSJ1, and MOGS. The intersection of these databases, visualized in a Venn diagram, revealed common miRNAs targeting COASY, FTSJ1, and MOGS. To further elucidate the intricate relationships between these genes, their regulatory miRNAs, and associated protein interactions, we constructed a PPI network using cytoscape. This network visualization provides insights into the complex regulatory mechanisms affecting the expression of COASY, FTSJ1, and MOGS and their potential impact on cellular processes. The analysis not only highlights the complex interactions between miRNAs and these genes but also establishes a foundation for investigating the role of these miRNAs in regulating gene expression in different physiological and pathological contexts (**Figure 9**).

## 4 Discussion

Lung adenocarcinoma (LUAD) continues to pose a significant global health challenge, as it is the leading cause of cancer-related deaths worldwide (37). This malignancy is predominantly influenced by risk factors including smoking, air pollution, and genetic predispositions. The high mortality rate associated with LUAD can largely be attributed to late-stage diagnosis, which significantly diminishes the efficacy of therapeutic interventions (3). Therefore, early detection and a deeper understanding of the molecular mechanisms underlying LUAD are crucial for enhancing patient outcomes and developing targeted therapies.

Our research used comprehensive data analysis from the GEO and TCGA databases, integrated with advanced bioinformatics tools including Weighted Gene Co expression Network Analysis (WGCNA) and Machine Learning, etc. Through this integrative approach, we identified key differentially expressed biomarkers (COASY, FTSJ1, and MOGS) to elucidate the molecular mechanisms of mitochondrial autophagy in LUAD and to identify potential therapeutic targets. This integrative approach promises to enhance early diagnosis and facilitate personalized treatment strategies, ultimately improving the prognosis for LUAD patients (38).

To enhance the efficiency and biological significance of the analysis, we conducted preliminary screening of differentially expressed genes (DEGs) prior to WGCNA analysis. This pre-screening step aims to reduce data dimensionality and computational complexity, thereby improving the biological effectiveness of subsequent network analysis. Although this method may introduce some bias, it allows us to concentrate on key genes associated with LUAD and mitochondrial autophagy, thus enhancing the biological interpretability of the results and the effectiveness of module detection.

Existing clinical biomarkers, such as adenosine deaminase (CEA) and cytokeratin (CYFRA21-1), have played a certain role in the early diagnosis of lung cancer. However, the specificity and sensitivity of these biomarkers are influenced by many factors, which may lead to false positive or false negative results. The new biomarkers in this study (COASY, FTSJ1, and MOGS) have certain advantages compared to them.

### 4.1 High specificity and sensitivity

COASY, as a coenzyme A synthase, plays a pivotal role in cellular metabolism, potentially serving as an important biomarker for early detection in LUAD patients (34). Our ROC curve analysis reveals an AUC value of 0.888 for COASY, suggesting its diagnostic potential in LUAD exceeds that of traditional biomarkers. FTSJ1, an enzyme involved in tRNA modification, has been shown to be overexpressed in various cancers, correlating with tumor malignancy (39). In our study, the AUC value for FTSJ1 was 0.883, indicating its significant diagnostic value in LUAD and potential association with tumor prognosis. MOGS, an endoplasmic reticulum glycosidase, exhibits overexpression in tumor cells, which is linked to abnormal glycoprotein modifications and may influence tumor invasiveness (40). The AUC value of MOGS is 0.859, highlighting its substantial diagnostic potential.

### 4.2 Multi cancer applicability

Through whole cancer analysis, we found that these three biomarkers also exhibit high expression levels in various cancers, indicating their potential importance not only for lung adenocarcinoma, but may also be valuable biomarkers in other types of cancer.

Mitochondrial autophagy is an important form of cellular autophagy that maintains cellular homeostasis by selectively degrading damaged or dysfunctional mitochondria (10). In this study, we identified COASY, FTSJ1, and MOGS as genes associated with mitochondrial autophagy, whichmay play important roles in the occurrence of LUAD. Especially through WGCNA analysis, we found that gene enrichment in the green-yellow module is associated with the FOXM1 pathway and retinoblastoma (RB) pathway, which play a central role in cancer cell cycle regulation, proliferation, and DNA repair. The FOXM1 pathway regulates the G1/S and G2/M transitions of the cell cycle, while the RB pathway controls the progression of the cell cycle and maintains normal cell proliferation and division by inhibiting the activity of E2F transcription factors (41). Dysregulation of these pathways has been shown to be a critical driver of cancer development, suggesting that targeting these pathways may represent a promising therapeutic strategy (41).

The immune microenvironment of LUAD plays a pivotal role in tumor progression and patient prognosis. This study also revealed the association between COASY, FTSJ1, and MOGS and immune cell infiltration, suggesting that these genes may affect the immune escape mechanism and therapeutic response of LUAD by regulating the immune microenvironment. Multiple studies have confirmed the critical role of immune cells in LUAD (42), with specific types of immune cells, such as CD8+ T cells and B cells, being strongly correlated with patient survival (43–47). Our research findings align with these observations, further indicating that COASY, FTSJ1, and MOGS may regulate the tumor microenvironment through interactions with immune cells, thereby impacting the prognosis of patients.In summary, the integration of immune cell infiltration analysis with gene expression profiling provides profound insights into the tumor microenvironment of LUAD. Our study contributes to the expanding body of evidence demonstrating that immune cell dynamics are integral to LUAD progression and treatment response.

Although this study reveals the potential of COASY, FTSJ1, and MOGS in LUAD, several limitations must be acknowledged. Firstly, the research data primarily originate from public databases such as GEO and TCGA. The heterogeneity of sample sources may introduce batch effects and selection bias, thereby compromising the accuracy and generalizability of the results. Additionally, the absence of detailed clinical information (e.g., treatment history and recurrence status) in these databases restricts a comprehensive evaluation of these genes’ clinical utility. Secondly, while pre-screening differentially expressed genes (DEGs) before WGCNA analysis effectively reduces data dimensionality, it may overlook genes with low expression or minimal changes, potentially affecting our understanding of the complex biological processes underlying LUAD. Thirdly, this study relies on bioinformatics analysis tools, and the interpretability and biological significance of its results are still limited. In particular, the mechanisms of action of COASY, FTSJ1, and MOGS at the molecular level are not yet clear and require further *in vitro* and *in vivo* experimental verification. Fourthly, this study lacks large-scale clinical validation, especially for different LUAD subtypes and patient populations, which limits the clinical applicability of the results. Although my research used ROC-AUC as the model performance evaluation criterion and achieved high prediction accuracy in the validation set, we have not yet conducted further generalization ability testing using methods such as k-fold cross validation. In addition, we have not yet reported key indicators such as accuracy, recall, F1 score, and MCC, which may limit the comprehensive evaluation of model performance to some extent. Fifthly, although our study cross validated the reliability of the selected biomarkers through multiple machine learning algorithms and demonstrated good robustness in the fusion and bioinformatics analysis of different datasets, we are aware that differences between datasets may affect the stability and predictive ability of the model. Therefore, further external dataset validation is crucial for improving the universality and clinical applicability of the model. Currently, due to time and resource constraints, we have not yet introduced new GEO or clinical datasets for additional validation. Sixth, although this study used Random Forest, LASSO, and SVM, which have good feature selection ability and interpretability in bioinformatics and biomarker screening tasks, we also recognize the potential value of other methods. For example, deep learning methods (such as MLP, CNNs) or enhanced decision tree methods (such as XGBoost), which can help to further verify the effectiveness of the selected methods.

Future research directions will focus on the following areas:

### 4.3 Clinical data validation

Conduct large-scale multicenter studies covering multiple samples of clinical data as well as external publicly available data, aiming to validate the practical application potential of COASY, FTSJ1, and MOGS in different LUAD subtypes and patient populations, thereby ensuring that the research results have broad clinical applicability and universality.

### 4.4 Mechanism research

Conduct in-depth molecular mechanism research to elucidate the mechanisms of COASY, FTSJ1, and MOGS in LUAD, with a focus on elucidating their roles in signaling pathways associated with cell proliferation, migration, and metastasis. Validate the functions of these genes using both *in vitro* and *in vivo* experimental approaches.

### 4.5 Comprehensive multi omics analysis

Integrating genomic, transcriptomic, proteomic, and metabolomic data, we perform a comprehensive multi-omics analysis to elucidate the intricate biological processes of LUAD and the roles of these biomarkers within these processes.

### 4.6 Machine learning model optimization

In machine learning analysis, we should further refine feature selection algorithms to improve the predictive performance of the model. In addition, the k-fold cross validation method is adopted and a more detailed hyperparameter adjustment strategy is provided to further enhance the robustness and generalizability of the model.

### 4.7 Personalized treatment strategy

Based on research into COASY, FTSJ1, and MOGS, this study investigates their associations with immune cell infiltration and the tumor microenvironment, aiming to identify novel targets and strategies for personalized LUAD treatment.

### 4.8 Discovery and application of new biomarkers

Expand the search for additional potential biomarkers, integrate machine learning and bioinformatics approaches to identify novel genes or pathways associated with LUAD, and assess their utility in early diagnosis and prognostic evaluation.

### 4.9 Further explore more advanced machine learning and deep learning methods to enhance the performance of models and the broad applicability of research

We will consider introducing XGBoost to further improve classification performance, while exploring the potential applications of deep learning methods such as MLP and CNNs on larger datasets. In addition, we also plan to optimize the hyperparameter tuning strategy of existing models and combine it with new datasets or hybrid model methods to improve the model’s generalization ability and stability. These improvement measures will help further enhance the effectiveness of the selected methods in biomarker recognition tasks, and improve the reliability and generalizability of research results.

## 5 Conclusion

This study investigates the significance and potential mechanisms of novel mitochondrial autophagy-related biomarkers COASY, FTSJ1, and MOGS in lung adenocarcinoma (LUAD). Through large-scale data analysis of GEO and TCGA public databases, combined with weighted gene co expression network analysis (WGCNA) and machine learning tools, differential expression of these genes in LUAD was identified, we identified differential expression patterns of these genes in LUAD. Our findings suggest that their specificity and sensitivity for early diagnosis and prognostic evaluation surpass those of existing clinical biomarkers. Furthermore, the critical roles of these genes in cell cycle regulation, mitochondrial autophagy, and immune microenvironment modulation indicate their potential as new therapeutic targets. Despite limitations such as data heterogeneity and lack of clinical validation, future research should focus on clinical data validation, in-depth mechanism studies, and multi omics collaborative analysis to promote personalized treatment of lung adenocarcinoma and the discovery of new biomarkers. Overall, this study provides a new perspective and strategic basis for the early diagnosis, mitochondrial autophagy research, and treatment of LUAD.

## Data Availability

The original contributions presented in the study are included in the article/**Supplementary Material**. Further inquiries can be directed to the corresponding author.
