# Supplementary figures and images for: The role and machine learning analysis of mitochondrial autophagy-related gene expression in lung adenocarcinoma
Source: Front Immunol. 2025 Apr 17;16:1509315. doi: 10.3389/fimmu.2025.1509315 (PMC12043613; doi:10.3389/fimmu.2025.1509315)

A

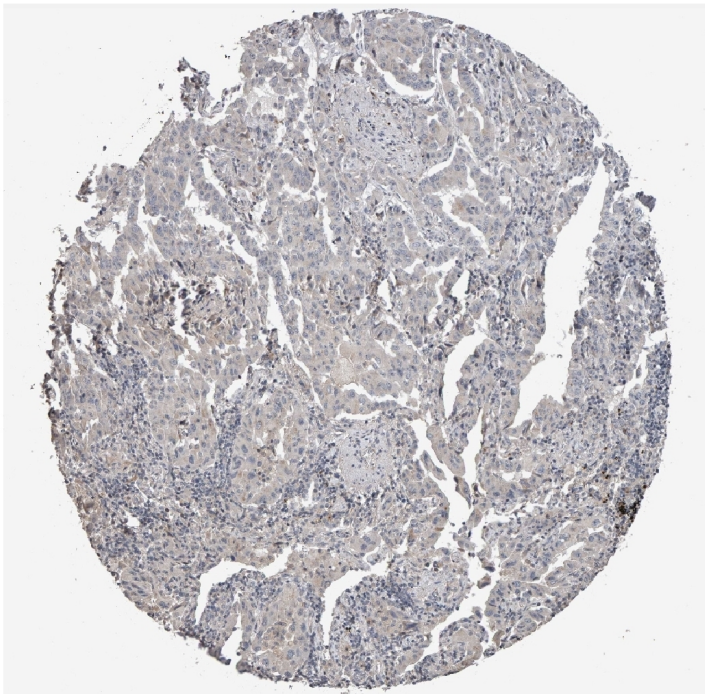

B

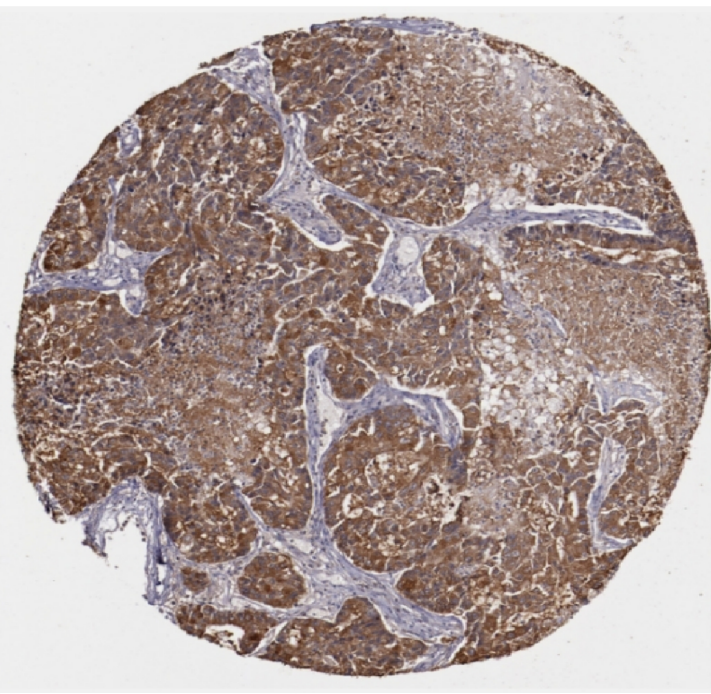

C

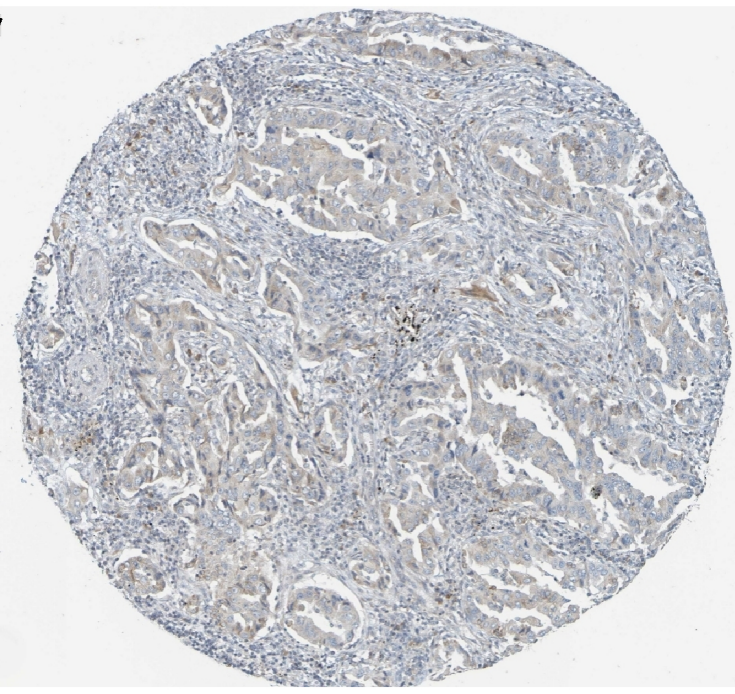

D

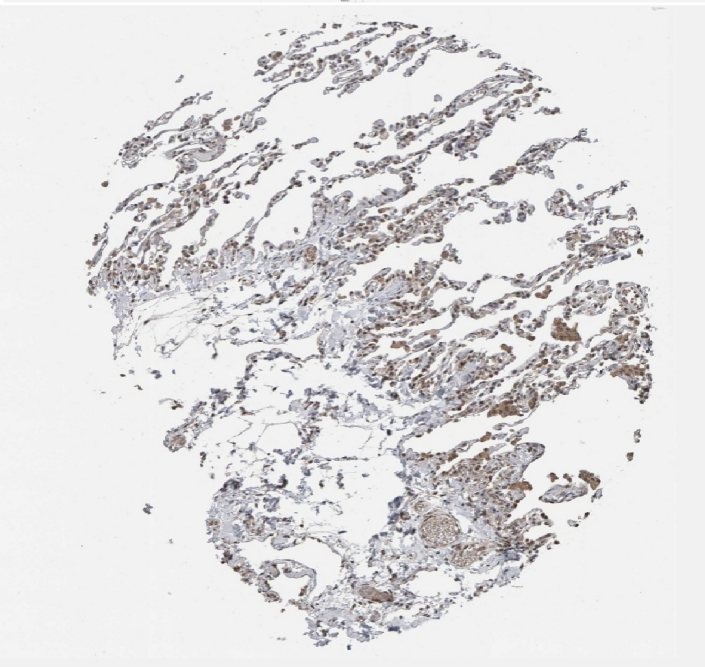

E

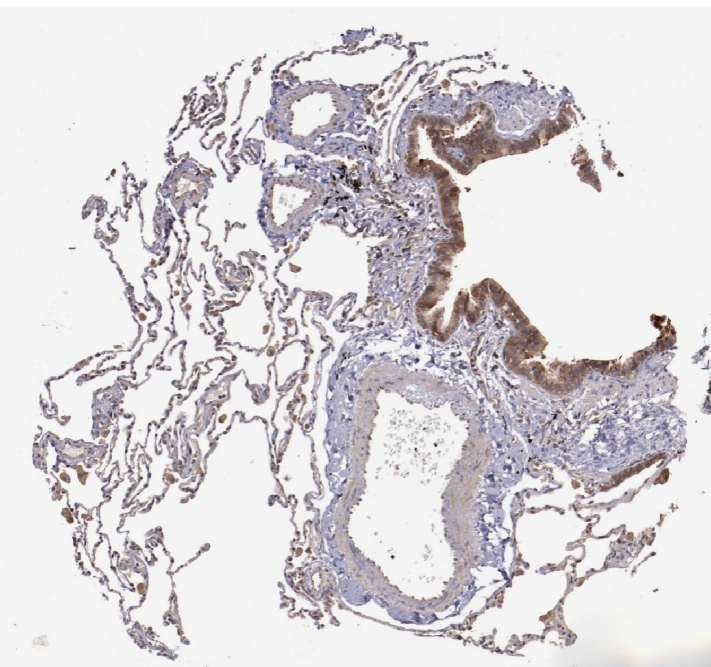

F

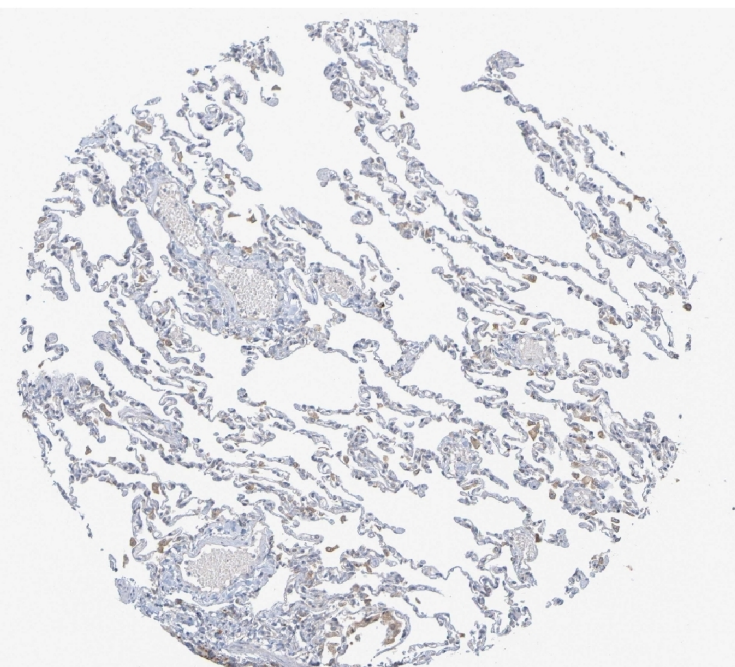

G

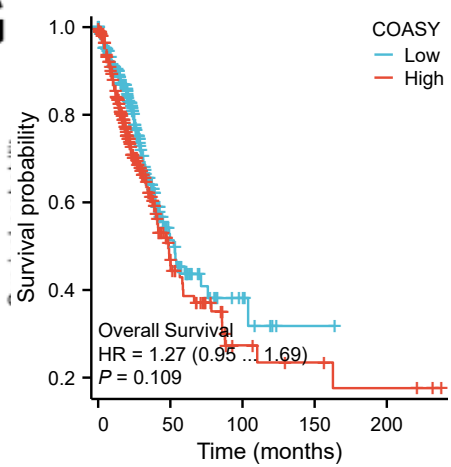

H

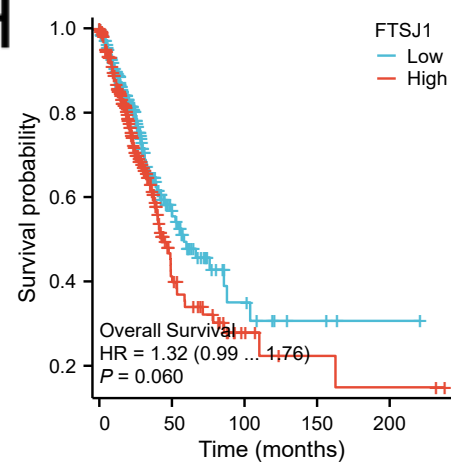

I

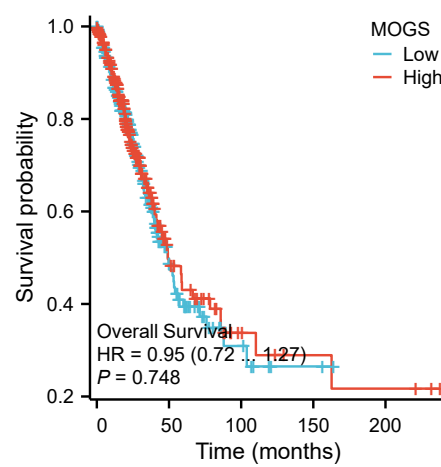

J

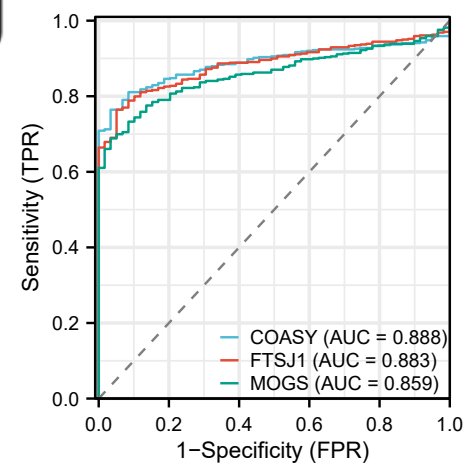

K

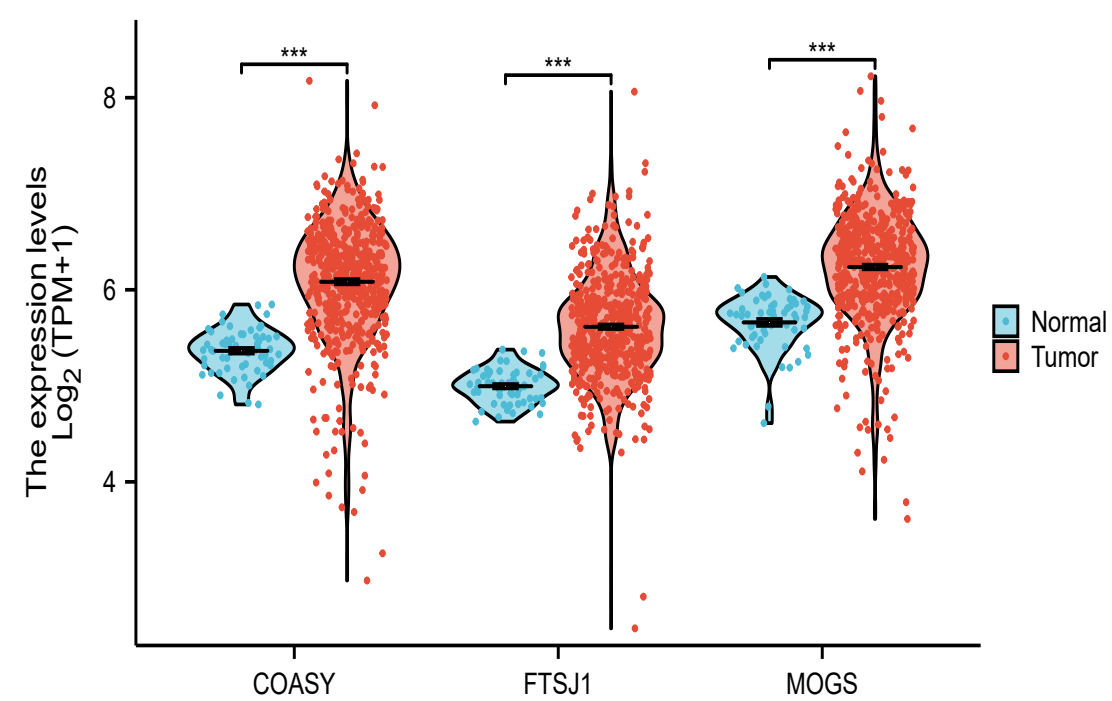

L

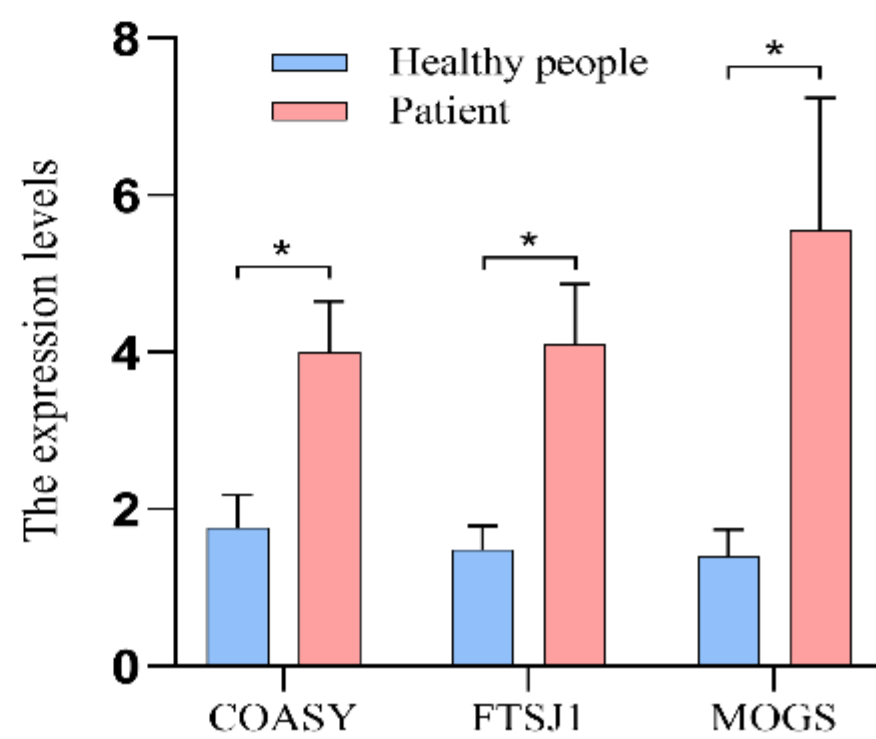

Supplement: Supplementary file 3 [file DataSheet3.pdf]

**A**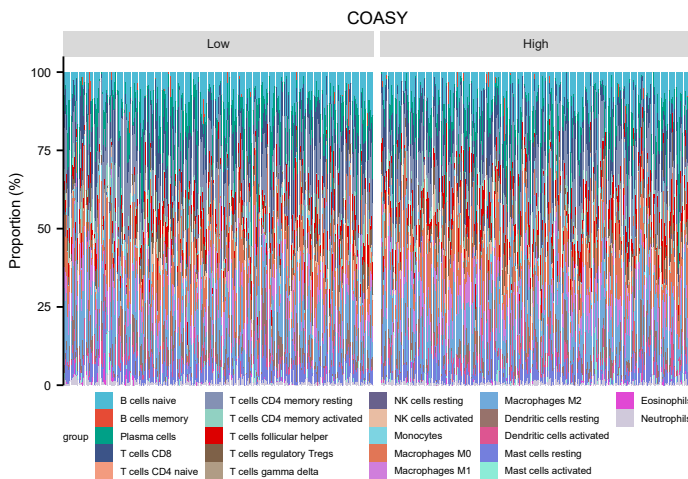**B**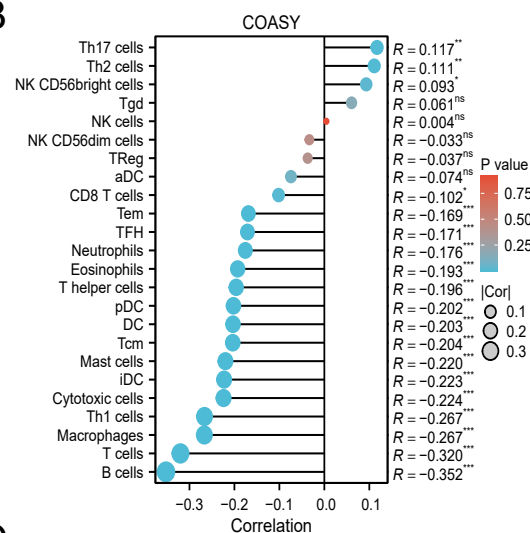**C**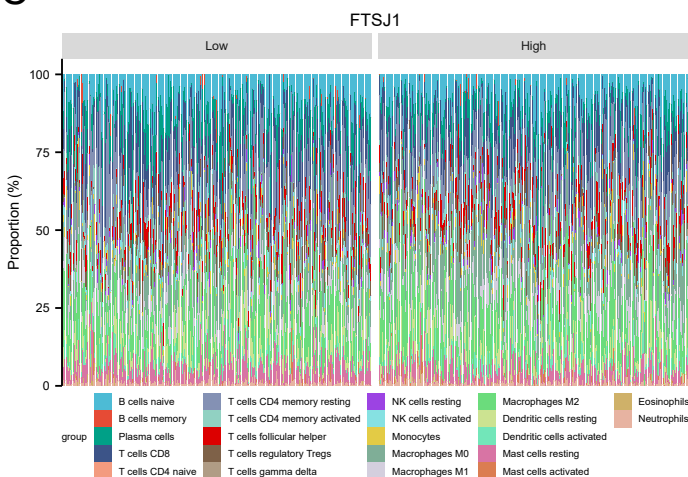**D**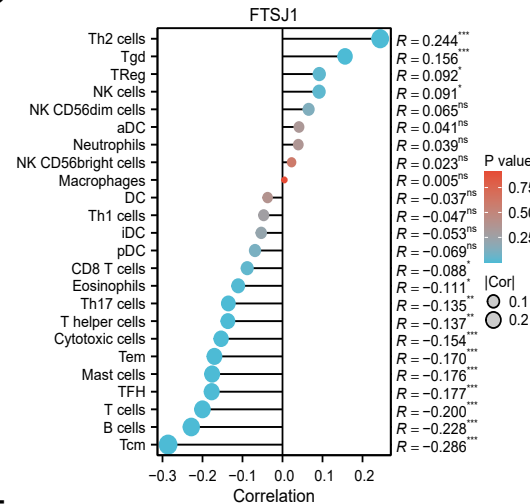**E**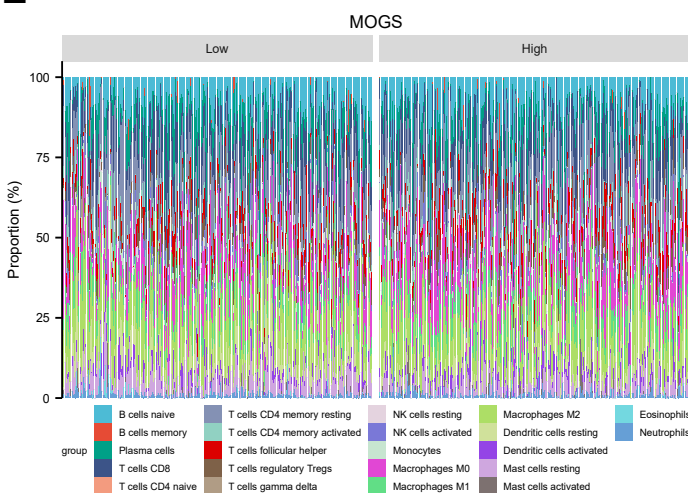**F**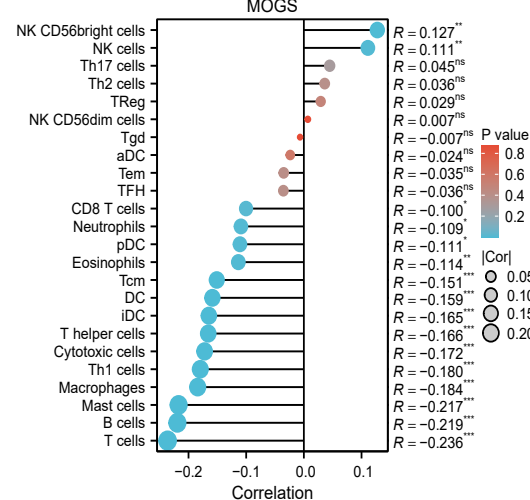

Supplement: Supplementary file 4 [file DataSheet4.pdf]
