# Supplementary material for: The role and machine learning analysis of mitochondrial autophagy-related gene expression in lung adenocarcinoma
Source: Front Immunol. 2025 Apr 17;16:1509315. doi: 10.3389/fimmu.2025.1509315 (PMC12043613; doi:10.3389/fimmu.2025.1509315)
Supplement: Supplementary file 2 [file DataSheet2.pdf]

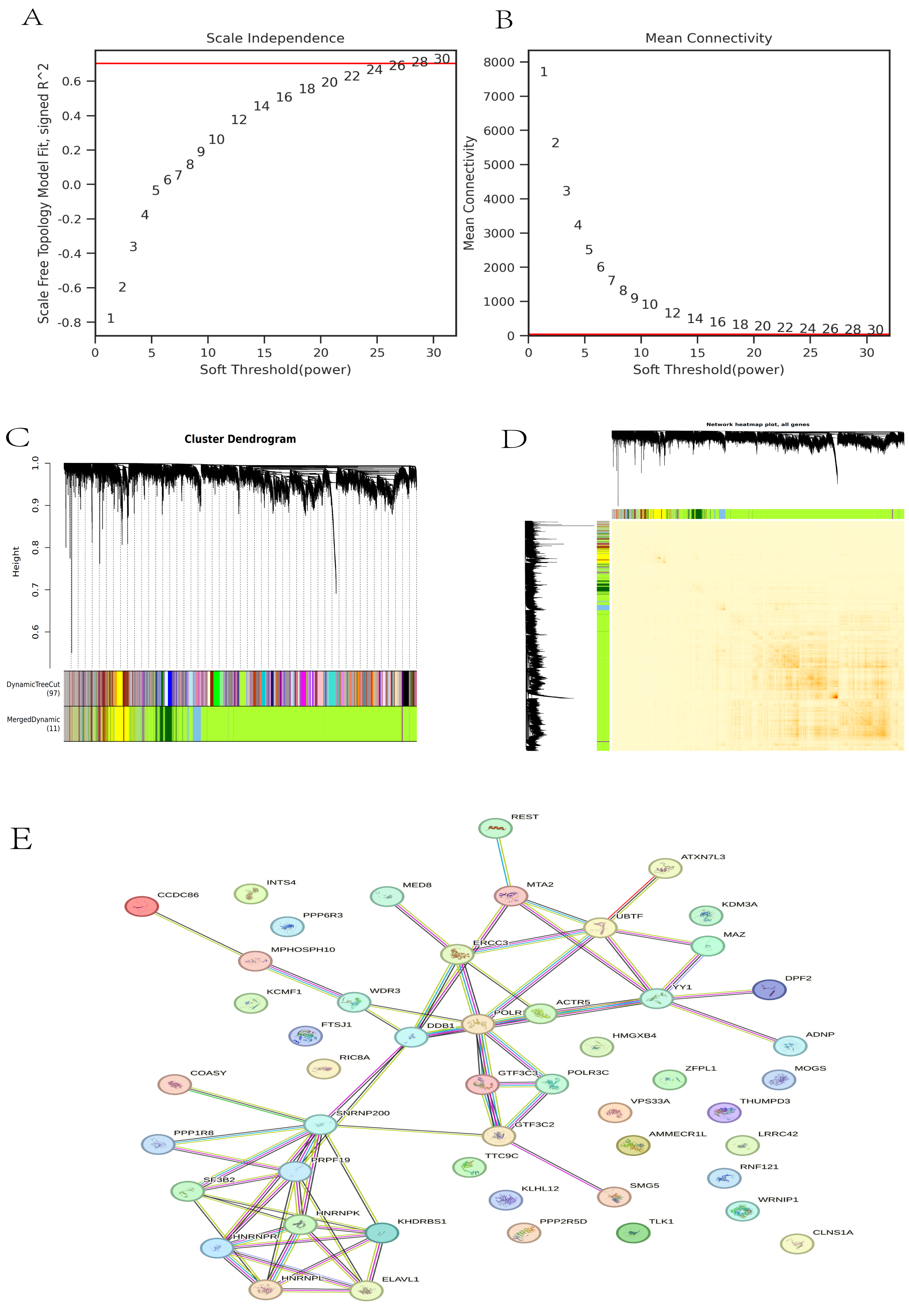
A
Scale Independence
Scale Free Topology Model Fit, signed R^2
Soft Threshold(power)
B
Mean Connectivity
Mean Connectivity
Soft Threshold(power)
C
Cluster Dendrogram
Height
DynamicTreeCut (97)
MergedDynamic (11)
D
Network heatmap plot, all genes
E
REST
ATXN7L3
INTS4
MED8
MTA2
CCDC86
KDM3A
PPP6R3
UBTF
MAZ
ERCC3
MPHOSPH10
DPF2
YY1
KCMF1
WDR3
ACTR5
POLR
FTSJ1
DDB1
ADNP
HMGXB4
RIC8A
ZFPL1
MOGS
COASY
GTF3C3
POLR3C
VPS33A
THUMPD3
SNRNP200
PPP1R8
GTF3C2
AMMECR1L
LRRC42
TTC9C
PRPF19
SF3B2
RNF121
KLHL12
SMG5
WRNIP1
HNRNPK
KHDRBS1
HNRNPR
PPP2R5D
TLK1
CLNS1A
HNRNPL
ELAVL1
